# Sex Differences in Double Poling Performance: The Role of Upper‐Body Strength and Endurance in Youth Elite Cross‐Country Skiers and Biathletes

**DOI:** 10.1002/ejsc.12253

**Published:** 2025-03-19

**Authors:** Carl‐Maximilian Wagner, John Owen Osborne, Øyvind Sandbakk, Stephan Schiemann, Daniel Röhrs, Tobias Schmidt, Michael Keiner

**Affiliations:** ^1^ Institute of Exercise, Sport and Health Leuphana University Lüneburg Lüneburg Germany; ^2^ School of Sport Science UiT The Artic University of Norway Tromsø Norway; ^3^ Sunshine Coast Hospital and Health Service Birtinya Australia; ^4^ School of Health University of the Sunshine Coast Sippy Downs Australia; ^5^ School of Exercise and Nutrition Sciences Queensland University of Technology Brisbane Australia; ^6^ Department of Sports Medicine Institute for Human Movement Science University of Hamburg Hamburg Germany; ^7^ MSH Medical School Hamburg Institute of Interdisciplinary Exercise Science and Sports Medicine Hamburg Germany; ^8^ Department of Sport Science German University of Health and Sport Ismaning Germany

**Keywords:** adolescents, endurance performance, fat‐free mass, maximal strength, power, vlamax

## Abstract

The study aimed to investigate sex differences in double poling (DP) ergometer performance among youth elite cross‐country skiers and biathletes and determine if these sex differences may be explained by upper‐body strength and endurance capacities. Thirteen female and nine male youth elite cross‐country skiers and biathletes (age: 16.7 ± 1.7 years; VO_2max_: 60.7 ± 6.3 mL·kg^−1^ min^−1^), matched for relative performance, completed a test battery evaluating upper‐body strength and power along with various endurance parameters on a DP ergometer. Testing included one‐repetition maximum (1RM) in upper‐body exercises, maximal oxygen uptake (VO_2max_) running test, and DP ergometer incremental test to exhaustion and sprint tests to determine peak oxygen uptake (VO_2peak_‐DP), maximal lactate accumulation rate (*v*La_max_), and power. Body mass and body composition were measured using bioelectrical impedance analysis. The findings demonstrated that the absolute differences in maximal strength, peak, and mean DP power outputs from both sprint and incremental tests to exhaustion (29%–38% difference), as well as maximal and peak oxygen uptake (29%–31%) between male and female athletes, were considerably reduced (2%–12%) following normalization to fat‐free mass (FFM). Correlations of absolute and FFM normalized VO_2max_, VO_2peak_‐DP with peak and mean power output from both sprint and incremental test to exhaustion during DP ergometry were significant (*r*_*xy*_ = 0.69–0.87) and remained consistent after correction for sex (*r*_*xy‐z*_ = 0.61–0.84). These findings suggest that sex performance differences are primarily attributable to absolute differences in maximal aerobic power, maximal strength, and FFM.

**Summary**

- The study identified significant sex‐based disparities in double poling (DP) performance, which were found to align with differences in maximal strength and aerobic capacity between male and female youth elite cross‐country skiers and biathletes.
- Normalization for fat‐free mass (FFM) largely attenuated these discrepancies. This finding suggests that the observed disparities in DP performance may be attributable to differences in FFM rather than to innate physiological sex differences, which in turn could influence the absolute values of strength and peak aerobic capacity.
- The smaller performance discrepancies observed in youth athletes within this study compared to older populations from previous studies, particularly when normalized for FFM, underscore the potential influence of training in attenuating sex‐based differences. Future research should investigate the extent to which systematic upper‐body strength training could further diminish disparities and enhance performance in both male and female athletes.

## 1 Introduction

Cross‐country skiing is an outdoor winter sport performed across varied terrain, which requires frequent transitions between sub‐techniques, involving coordinated efforts from both upper and lower extremities (Sandbakk et al., 2011a; Sandbakk, Holmberg 2014). Achieving competitive success in cross‐country skiing requires a high maximal oxygen uptake (VO_2max_), high fractional utilization of VO_2max_, and efficient energy conversion (i.e., gross efficiency [GE]), combined with robust anaerobic capacity, maximal strength and power in addition to the technical, tactical, and mental aspects of skiing performance (Sandbakk, Holmberg 2014; Hébert‐Losier et al. 2017; di Prampero 2003; Sandbakk et al., 2011b). The observed increases in speeds observed in competitive events in recent years (Stöggl and Stöggl 2013; Losnegard 2019) have resulted in an increasing importance being placed on high‐speed techniques such as G3 skating and double poling (DP). As propulsion in these techniques, particularly DP (Holmberg et al., 2005; Holmberg, 2005), relies heavily on poling (Sandbakk, Ettema, and Holmberg 2014b; Stoggl et al. 2011), they demand substantial upper‐body strength and power to generate high peak forces within short contact times, thereby increasing cycle length, and consequently, maximal velocities (Stoggl et al. 2011; Sunde et al. 2019; Alsobrook et al. 2009). Over the past 4 decades, athletes have notably improved the ratio between peak oxygen uptake (VO_2peak_) during upper‐body exercise and VO_2max_, which has increased from approximately 70%–95%. Although the literature generally reports variability in this ratio between 5% and 15%, contemporary elite athletes strive to achieve peak oxygen uptake levels during DP (VO2peak‐DP) of ≥ 95% VO2max to optimize performance (Holmberg, 2005; Stöggl et al., 2019).

While male cross‐country skiers typically outperform their female counterparts by 10%–15% (Sandbakk, Solli, and Holmberg 2018; Andersson et al. 2019; Seiler, De Koning, and Foster 2007), performance differences between sexes increase as the contribution from poling also increases. The absolute peak power differences of 67% in DP are significantly larger than the 58%–62% observed in whole‐body movements, such as G3 skating and diagonal striding, though normalization for body mass (BM) and fat‐free mass (FFM) reduces these differences to 3%–20% (Sandbakk, Ettema, and Holmberg 2014b). DP ergometry has shown absolute mean power differences as high as 87% during a self‐pace 3‐min performance test (Hegge et al. 2016). Concurrently, male athletes exhibit significantly higher VO_2peak_ (51%–63%) and higher fractional utilization of VO_2max_ (3%–10%) during DP (ergometry) compared to females (Sandbakk, Ettema, and Holmberg 2014b; Hegge et al. 2016). Previous research indicates that during upper‐body dominant exercises, absolute power and VO_2peak_ are significantly influenced by active muscle mass (i.e., recruited muscle mass at work) (Astrand and Saltin 1961; Shephard et al. 1988), leading to greater sex differences in upper‐body exercise modalities compared to lower‐body exercise modalities due to males' relatively larger upper‐body muscle mass (Sandbakk, Ettema, and Holmberg 2014b; Hegge et al. 2015, 2016; Janssen et al. 2000). These sex discrepancies are attributed to biological factors, including higher testosterone hemoglobin levels, greater muscle mass, and lower‐body fat in males, subsequently enhancing the delivery of aerobic and anaerobic energy, and maximal strength, even at relative performance levels (Sandbakk, Solli, and Holmberg 2018; Joyner 2017).

A recent study by Sollie and Losnegard (Sollie and Losnegard 2022) found that the discrepancy in performance between male and female elite adolescent skiers, aged 14–15 years, was analogous to that observed in elite junior (approximately 18 years) and world‐class senior cross‐country skiers, when matched for performance within age‐groups. The study, however, concentrated exclusively on treadmill roller ski skating, with a particular focus on the G2 sub‐technique. Consequently, while sex differences in performance are widely recognized in adult athletes, there is still a lack of research relating to sex differences in youth cross‐country skiers, particularly in upper‐body strength, endurance capacity, and DP performance. Furthermore, there is a paucity of data addressing how sex disparities in maximal strength and upper‐body endurance capacity influence performance in exercises that predominantly engage the upper body.

To develop effective and individualized training programs, it is imperative to investigate the relative importance of upper‐body strength and endurance capacity and their associations with performance, both independent and dependent of sex. Therefore, the primary objective of this study was to investigate sex differences in DP ergometer performance among youth elite cross‐country skiers and biathletes. A secondary objective was to determine how these sex differences could be explained by upper‐body strength and endurance capacities.

## 2 Methods

### 2.1 Participants

Twenty‐two (male, *n* = 9; female, *n* = 13) national‐level (Tier 3, (McKay et al. 2022)) youth cross‐country skiers (*n* = 8) and biathletes (*n* = 14) from two elite training centers volunteered to participate in this study. The participants were consistently ranked among the top 30 in their respective national and international competition classes, based on their average placements across all reported competitions. This placed them in the category of elite athletes (Lorenz et al. 2013). The group's average seasonal placement was 17th, with individual race rankings ranging from 1st to 52nd. The mean ± standard deviation (SD) (95% confidence interval [CI]) characteristics of the group were as follows: age: 16.7 ± 1.7 (16.0–17.5) years; body height: 1.70 ± 0.09 (1.66–1.74) m; BM: 58.9 ± 8.0 (55.3–62.5) kg; body mass index (BMI): 20.3 ± 1.7 (19.5–21.0) kg·m^−2^; body fat: 16.5 ± 6.4 (13.6–19.3) %; FFM: 49.3 ± 8.7 (45.5–53.2) kg; and VO_2max_: 3588 ± 728 (3265–3910) mL·min^−1^ and 60.7 ± 6.3 (57.9–63.5) mL·kg^−1^ min^−1^. Table 1 provides an overview of the training characteristics of the participants over the last 3 months prior to the data collection (September to December), based on participants' training diaries), presented as mean ± SD (CI). The majority of participants, with the exception of the five youngest individuals, reported systematic resistance training experience, ranging from 6 months to several years. Fifteen athletes had completed a 10‐week high‐volume upper‐body resistance training block, comprising two weekly training sessions with 10 sets per muscle group (Wagner et al. 2024). All participants performed regular core stability training, incorporating band or sling exercises, Swiss Ball routines, and various mat exercises, targeting lower back and abdominal muscles. Each subject and their parents (if the subjects were younger than 18 years) were informed about the aims of the study and the experimental risks involved with the research before providing written informed consent. Furthermore, this study was performed in accordance with the Declaration of Helsinki and was approved by the Universities Ethics Committee (DHGS‐EK‐2023‐004).

**TABLE 1:** Descriptive data of 3‐months’ weekly training volume and training intensity distribution (September to December) in a group of male and female cross‐country skiing and biathlon athletes. Data are presented as mean ± SD (CI).

Endurance training in weekly hours			
	All (*n* = 22)	Male (*n* = 9)	Female (*n* = 13)
Zone 3	0.9 ± 0.1 (0.8–1.0)	0.9 ± 0.2 (0.7–1.0)	0.9 ± 0.1 (0.8–1.0)
Zone 2	0.3 ± 0.1 (0.3–0.4)	0.3 ± 0.1 (0.2–0.4)	0.3 ± 0.2 (0.2–0.4)
Zone 1	10.2 ± 1.0 (9.8–10.7)	10.5 ± 1.1 (9.7–11.4)	10.0 ± 0.9 (9.4–10.6)
Total	11.4 ± 1.0 (11–11.9)	11.7 ± 1.2 (10.8–12.6)	11.2 ± 0.9 (10.7–11.8)

*Note:* Zone 1%, 55%–82% of HR_max_; zone 2%, 82%–87% of HR_max_; zone 3%, 87%–97% of HR_max_ based on a physiological 3‐zone intensity model, anchored around ventilatory/lactate thresholds 1 and 2 (Sylta, Tønnessen, and Seiler 2014).

### 2.2 Experimental Design

Conducted over two consecutive days, the study involved a series of tests evaluating both upper‐body strength and maximal aerobic power (Day 1), along with various endurance parameters on a DP ergometer (Day 2). The assessment protocol comprised a maximal sprint to determine the participants' maximal lactate accumulation rate (*v*La_max_) and an incremental test to exhaustion. Verbal encouragement was provided to athletes throughout all tests to stimulate maximal efforts. All participants were familiar with the testing protocols from familiarization sessions and/or training experience (Wagner et al. 2024). To minimize potential confounding factors, participants were asked to limit their exercise to a maximum of 90 minutes at low intensities (< 75% of maximum heart rate [HR_max_]) the day before the testing. Participants were instructed to avoid consuming large meals or coffee or other products containing caffeine during the 3 h prior to testing. On test days, participants refrained from performing any training before testing. All tests were conducted under similar environmental conditions, specifically at a temperature range of 21°C–24°C and at approximately the same time of day, within a range of ± 1.5 h, to avoid circadian variance.

### 2.3 Assessment of Anthropometric Characteristics

#### 2.3.1 Bioelectrical Impedance Analysis

Data collection procedures were conducted following previously described methods (Schoenfeld et al., 2020). The participants were given specific instructions, including refraining from eating for 8 h prior to testing, minimizing fluid intake on the morning of the test and emptying the bladder immediately before the test. Anthropometric characteristics were assessed using a measuring tape for standing body height. BM and body composition, including total fat‐free mass and fat mass, were analyzed using a bioelectrical impedance analysis device (InBody MC‐780MA‐N; InBody, Cerritos, CA, USA).

### 2.4 Assessment of Upper‐Body Maximal Strength

#### 2.4.1 One‐Repetition Maximum

Upper‐body maximal strength was assessed using the one‐repetition maximum (1RM) in bench press and bench pull. Repetition maximum testing was conducted in accordance with the guidelines established by the National Strength and Conditioning Association (Haff and Triplett 2016). The warm‐up protocol included a 10‐min warm‐up on a cycle ergometer, followed by 3 sets with 2–5 repetitions at approximately 50%–80% of 1RM for each exercise. The initial attempts were performed with a load of approximately 90%–95% of the estimated one‐repetition maximum (1RM). The load was then increased by 2%–5% after each successful attempt until the participants were unable to lift or pull the load with proper technique. The highest accepted weight after two consecutive nonaccepted attempts was registered as the 1RM. Rest periods of at least 5 minutes were given between the trials. 1RMs were achieved within a maximum of five attempts (bench press: 4 ± 1; bench pull: 4 ± 1). All 1RM testing was supervised by the same investigator and conducted on the same equipment with identical equipment positioning for each subject. The testing order for bench press and bench pull was the same in all testing sessions.

Bench press performance was tested in supine position on a bench. A five‐point body contact position (head, upper back, and buttocks firmly on the bench with both feet flat on the floor) was required to be considered proper form. During the eccentric phase of the exercise, a gentle contact of the barbell with the chest was permitted, although the attempt was considered unsuccessful if the chest movement helped the execution. The end position was determined by fully extended elbows at the end of the concentric movement (Schoenfeld et al., 2016).

Bench pull performance was tested in a prone position on a bench. The examiner visually inspected whether the arms were straight as participants grabbed the barbell. The athletes performed a concentric arm flexion beginning from the extended position. The end position was determined by touching the bench with an elbow angle of ≤ 90° (Akça 2014). Chest and lower extremities were required to stay in contact with the bench for a trial to be successfully performed.

Given the significance of BM for endurance performance, 1RMs were reported as both absolute (kilogram) and relative strength (kilogram per kilogram of BM).

### 2.5 Assessment of Maximal Aerobic Power

#### 2.5.1 Maximal Oxygen Uptake Test

Following a standardized 20‐min warm‐up (60%–80% of HR_max_), the subjects ran with poles on a treadmill at a constant incline of 10.5%. The speed increased incrementally by 1 km·h^−1^ every 60 s until exhaustion, with a starting speed of 7 km·h^−1^. The subjects reached exhaustion within 4:45 to 8:15 min. To evaluate whether VO_2max_ was achieved, two or more of the following criteria had to be met: (1) a plateau in oxygen uptake, observed as a leveling off of VO_2_ versus rate of work, with VO_2_ on the highest work rate being within 150 mL·min^−1^ from the previous highest obtained value in the test (Brink‐Elfegoun et al. 2007), (2) a respiratory exchange ratio (RER) greater than 1.10, (3) heart rate (HR) greater than 95% of the subjects' reported HR_max_, or (4) blood lactate concentration ([La‐]) greater than 8.0 mmol·L^−1^ (Sandbakk et al. 2010). Continuous VO_2_ measurements were taken, and VO_2max_ was determined from the average of the three highest 10‐s consecutive measurements. The highest HR averaged over 30 s during the test was considered the subject's HR_max_ (Polar H10, Polar, Kempele, Finland). Peak lactate concentration ([La^−^]_peak_) was measured 1.5 min after completing the test. To calculate the maximal work rate (*P*_max_), power used against gravity (Pg) was calculated as the increase in potential energy per unit time (Pg = m·g·sin (*α*)·*v*, where *v* represents the belt speed and *α* represents the angle of incline. Peak power output (*P*_max_) was calculated using the aforementioned equation (Kuipers et al. 1985):

$$P_{\max}=P_{\text{COM}}+\left(t/60\right)\;\cdot \;W,$$

where *P*_COM_ is the power output of the last completed stage, *t* is the number of seconds completed in the last uncompleted stage, and *W* is the change in power output of the last uncompleted stage (Alsobrook et al. 2009).

Respiratory parameters (VO_2_, VCO_2_) were measured (10‐s sampling time) using a computerized metabolic unit with the mixing chamber (K5, Cosmed, Rome, Italy). The flow turbine was calibrated with a 3‐L calibration syringe. The metabolic system was calibrated with known concentrations of certified calibration gases before each test according to the specifications of the manufacturer. The spiroergometric system used for the DP tests was identical.

### 2.6 Assessment of Double Poling Capacity

A specially designed DP ergometer (SkiErg, Concept 2, Morrisville, VT) was used for all DP capacity tests. Power is generated by pulling cords that spin a wind resistance flywheel. The deceleration rate of the flywheel (drag factor) is controlled by a damper located on the flywheel. Therefore, the work required to accelerate the flywheel during each stroke is predetermined. For all performance tests, the damper settings were set at 100% of body weight to account for the influence of body weight for endurance performance. Similar setups were utilized in previous studies (Faiss et al. 2015). Participants were instructed to choose a position that mimicked their habitual positions during DP on skis.

Given the significance of body weight for endurance performance, mean power was reported in both absolute (Watts) as well as normalized values (Watts per kilogram of BM).

#### 2.6.1 Double Poling Power and Maximal Rate of Lactate Accumulation

After a self‐paced 5‐min warm‐up (approx. 1 W·kg^−1^ BM) on the ski ergometer, participants rested passively in a seated position for 5 min. Thereafter participants performed a 15‐s bout with maximal efforts to determine their maximal double poling power (DPP_15s_). The start and end of the sprint were communicated verbally using a countdown. Throughout all tests, researchers provided strong verbal encouragement to ensure participants exerted maximal efforts. The mean power was used for subsequent analyses. Participants were allowed to choose their own cadence. Wattage and cycle rate were recorded using software (C2 ErgData, Concept 2, Morrisville, VT).

Capillary blood lactate samples were obtained from the right earlobe immediately before the sprint test (EKF Biosen C‐line analyzer, Barleben, Germany) to establish resting lactate levels ([La^−^]_rest_). Post‐sprint, capillary blood samples were collected at 1‐min intervals to determine [La^−^]_peak_ with participants resting passively in a seated position until two samples in a row showed lower lactate values than the previous one. *v*La_max_ was calculated according to the following equation (Heck, Schulz, and Bartmus 2003):

$$v{\text{La}}_{\max}\left(\text{mmol}\;L^{-1}\;s^{-1}\right)=\left({\left[{\text{La}}^{-}\right]}_{\text{peak}}-{\left[{\text{La}}^{-}\right]}_{\text{rest}}\right)\cdot {\left(t_{\text{exerc}}-t_{\text{PCr}}\right)}^{-1}$$

where Δ[La^−^] between [La^−^]_rest_ and [La^−^]_peak_ is divided by the difference between the total exercise time (15 s) and the time contributed by the phosphagen system. In this equation, all terms except t_PCr_ are determined experimentally. t_PCr_ represents a modeled exercise period during which no [La^−^] formation hypothetically occurs, as energy needs are covered by the phosphagen system. With reference to Heck, Schulz, and Bartmus (Heck, Schulz, and Bartmus 2003), t_PCr_ was set to 3.5 s for all participants since the sprints lasted for 15 s.

#### 2.6.2 Peak Oxygen Uptake Test During Double Poling

After 5 min of rest, participants performed an incremental test to exhaustion on the DP ergometer. The test began at 75 W for women and 80 W for men, with the work rate increasing by 15 and 20 W, respectively, every minute. Exhaustion was defined as a power output drop of ≥ 5 W below the required work rate for five or more seconds. Participants were free to choose their own cadences at each stage of the test. The subjects reached exhaustion within 3:07 to 7:10 min. Cardiorespiratory variables were continuously monitored during the test. The highest average VO_2_ during a continuous 30‐s period was defined as VO_2peak_‐DP. The participants' peak heart rate during DP (HR_peak‐_DP) was determined as the highest HR averaged over 30 consecutive seconds (Polar H10, Polar, Kempele, Finland). [La‐]_peak_ was measured 1.5 min after completing the test. Peak power output (DPP_PEAK_) was calculated using an equation originally developed for stationary cycle ergometer test protocols (Kuipers et al. 1985): DPP_PEAK_ = DPP_COM_ + (t/60) × W. Here, DPP_COM_ is the power output of the last completed stage, *t* is the number of seconds completed in the last uncompleted stage, and W is the change in power output of the last uncompleted stage (Alsobrook et al. 2009).

### 2.7 Statistical Analysis

Statistical analyses were performed using SPSS 29.0.1.1 (IBM, Ehningen, DE, Germany). The best performance values were used in the data analyses for all performance tests. Data are presented as the means, standard deviations, and 95% confidence intervals. The level of significance was set a priori at *p* < 0.05 for all statistical methods. The Shapiro–Wilk test showed that all parameters were normally distributed, except for absolute *P*_max_ during treadmill running. Independent sample *t*‐tests were used to compare sex differences. Paired‐sample *t*‐tests were used to compare between upper‐ and lower‐body performance differences. The magnitude of the difference between variables was calculated using Cohen’s *d* effect size. In general, effect sizes of Cohen’s *d* defined as *d* > 0.8 can be interpreted as large effects, *d* > 0.5 can be interpreted as medium effects, and > 0.2 can be interpreted as small effects (Cohen 2013). The relationship between upper‐body strength and power along with various endurance parameters and DP ergometer performances was analyzed with one‐tailed bivariate Pearson correlation coefficient (*r*). To address the potential impact of sex on the strength of correlation coefficients, partial correlation analyses were conducted by controlling for sex. The correlation coefficient in standard correlations was denoted as *r*_xy_, whereas the correlation coefficient in partial correlations adjusted for sex was denoted as *r*_xy‐z_. Correlations were interpreted according to the following thresholds: ≤ 0.1 = trivial, > 0.1–0.3 = small, > 0.3–0.5 = moderate, and > 0.5 = large (Cohen 2013). Additionally, 95% CI for correlation coefficients were reported.

## 3 Results

The male participants exhibited significantly higher strength and power outputs, both absolute (29%–36%) and normalized to BM (15%–20%), compared with their female counterparts. However, the differences diminished through normalization to FFM (2%–7%, see Table 2). A comparable pattern was evident for VO_2max_ and VO_2peak_‐DP, where normalization to FFM resulted in a notable reduction in the observed sex differences (from 29%‐31% to 11%–12%). The study found no statistically significant differences in vLamax between male and female subjects, yielding a combined group mean of 0.27 ± 0.08 mmol·L^−1^ s^−1^ (range: 0.23–0.30 mmol·L^−1^ s^−1^).Moreover, there were no sex‐dependent differences in physiological measures of exertion (i.e., [La^−^]_peak_, HR_max_, RER) during the incremental tests (*p* = 0.176–0.973).

**TABLE 2:** Descriptive data of anthropometrics, performance, and physiological indices during strength tests, maximal endurance tests, as well as upper‐body anaerobic capacity tests in a group of male (*n* = 9) and female (*n* = 13) elite youth cross‐country skiing and biathlon athletes. Data are presented as mean ± SD [CI].

Variable	Sex	Mean	*t*‐Test	Effect size (%Difference)
Anthropometrics (bioelectrical impedance analysis)				
Body mass (kg)	♀	55.82 ± 6.14 [52.11–59.54]	T_20_ = −2.390	*d* = 1.036 (14%)
	♂	63.33 ± 8.64 [56.69–69.98]	*p* = 0.027*	
Fat‐free mass (kg)	♀	44.17 ± 4.73 [41.31–47.02]	T_20_ = −4.750	*d* = 2.060 (29%)
	♂	56.80 ± 7.78 [50.82–62.78]	*p* < 0.001*	
Body fat (%)	♀	20.8 ± 4.3 [18.2–23.4]	T_20_ = 6.312	*d* = 2.737 (10.5%‐point)
	♂	10.3 ± 2.9 [8.1–12.6]	*p* < 0.001*	
Maximal aerobic power and capacity (treadmill running with poles)				
P_peak_ (W)	♀	181.46 ± 20.57 [169.03–193.89]	T_20_ = −4.207	*d* = 1.824 (33%)
	♂	241.56 ± 45.58 [206.52–276.59]	*P* < 0.001*	
P_peak_·BM^−1^ (W·kg^−1^)	♀	3.25 ± 0.20 [3.12–3.37]	T_20_ = −5.145	*d* = 2.231 (17%)
	♂	3.80 ± 0.30 [3.57–4.03]	*p* < 0.001*	
P_peak_·FFM^−1^ (W·kg^−1^)	♀	4.12 ± 0.30 [3.94–4.30]	T_20_ = −0.897	*d* = 0.386 (3%)
	♂	4.23 ± 0.26 [4.03–0.43]	*p* = 0.380	
VO_2max_ (mL·min^−1^)	♀	3186.08 ± 331.21 [2985.9–3386.23]	T_20_ = −3.621	*d* = 1.792 (31%)
	♂	4168.56 ± 765.92 [3579.82–4757.29]	*p* = 0.005*	
VO_2max_·BM^−1^ (mL·kg^−1^· min^−1^)	♀	57.29 ± 3.73 [55.03–59.54]	T_20_ = −4.056	*d* = 1.759 (15%)
	♂	65.67 ± 5.99 [61.06–70.27]	*p* = 0.001*	
VO_2max_·FFM^−1^ (mL·kg^−1^ min^−1^)	♀	70.25 ± 10.56 [63.87–76.63]	T_20_ = −1.493	*d* = 0.647 (11%)
	♂	78.16 ± 14.35 [67.12–89.19]	*p* = 0.151	
Upper‐body aerobic power and capacity (double poling ergometry)				
DPP_peak_ (W)	♀	138.15 ± 25.95 [122.47–153.83]	T_20_ = −3.742	*d* = 1.623 (33%)
	♂	184.22 ± 31.71 [159.85–208.59]	*p* = 0.001*	
DPP_peak_·BM^−1^ (W·kg^−1^)	♀	2.47 ± 0.33 [2.27–2.66]	T_20_ = −3.209	*d* = 1.391 (18%)
	♂	2.91 ± 0.32 [2.67–3.16]	*p* = 0.004*	
DPP_peak_·FFM^−1^ (W·kg^−1^)	♀	3.14 ± 0.72 [2.70–3.58]	T_20_ = −0.685	*d* = 0.298 (7%)
	♂	3.35 ± 0.69 [2.82–3.88]	*p* = 0.501	
VO2_peak_‐DP (mL·min^−1^)	♀	2714.46 ± 298.70 [2533.96–2894.96]	T_20_ = −4.578	*d* = 1.985 (29%)
	♂	3509.44 ± 516.83 [3112.17–3906.72]	*p* = 0.000*	
VO2_peak_‐DP·BM^−1^ (mL·kg^−1^ min^−1^)	♀	48.37 ± 4.97 [45.37–51.37]	T_20_ = −3.171	*d* = 1.375 (15%)
	♂	55.66 ± 5.76 [51.23–60.08]	*p* = 0.005*	
VO2_peak_‐DP·FFM^−1^ (mL·kg^−1^ min^−1^)	♀	59.68 ± 10.14 [53.56–65.81]	T_20_ = −1.481	*d* = 0.643 (12%)
	♂	66.67 ± 11.89 [57.53–75.80]	*p* = 0.154	
Upper‐body anaerobic capacity test (double poling ergometry)				
DPP_15s_ (W)	♀	204.92 ± 50.18 [174.60–235.25]	T_20_ = −2.806	*d* = 1.217 (31%)
	♂	269.00 ± 55.08 [226.66–311.34]	*p* = 0.011*	
DPP_15s_ ·BM^−1^ (W·kg^−1^)	♀	3.64 ± 0.57 [3.30–3.98]	T_20_ = −2.704	*d* = 1.173 (16%)
	♂	4.23 ± 0.43 [3.90–4.57]	*p* = 0.014*	
DPP_15s_ ·FFM^−1^ (W·kg^−1^)	♀	4.61 ± 1.19 [3.89–5.33]	T_20_ = −0.604	*d* = 0.263 (7%)
	♂	4.93 ± 1.26 [3.97–5.90]	*p* = 0.552	
*v*La_max_ (mmol·L^−1^ s^−1^)	♀	0.25 ± 0.09 [0.20–0.31]	T_20_ = −0.837	*d* = 0.363 (16%)
	♂	0.29 ± 0.08 [0.23–0.35]	*p* = 0.412	
Strength tests (one‐repetition maximum)				
1RM bench pull (kg)	♀	46.15 ± 10.03 [40.09–52.22]	T_20_ = −3.328	*d* = 1.443 (31%)
	♂	60.56 ± 9.90 [52.94–68.17]	*p* = 0.003*	
1RM·BM^−1^ bench pull (kg·kg^−1^)	♀	0.82 ± 0.11 [0.76–0.89]	T_20_ = −3.907	*d* = 1.485 (17%)
	♂	0.96 ± 0.05 [0.92–0.99]	*p* = 0.001*	
1RM·FFM^−1^ bench pull (kg·kg^−1^)	♀	0.87 ± 0.17 [0.76–0.97]	T_20_ = −0.913	*d* = 0.342 (6%)
	♂	0.92 ± 0.10 [0.85–1.00]	*p* = 0.326	
1RM bench press (kg)	♀	38.65 ± 10.54 [32.29–45.02]	T_20_ = −3.217	*d* = 1.395 (36%)
	♂	52.50 ± 8.93 [45.64–59.36]	*p* = 0.004*	
1RM·BM^−1^ bench press (kg·kg^−1^)	♀	0.69 ± 0.13 [0.61–0.76]	T_20_ = −2.954	*d* = 1.281 (20%)
	♂	0.83 ± 0.09 [0.76–0.90]	*p* = 0.008*	
1RM·FFM^−1^ bench press (kg·kg^−1^)	♀	1.04 ± 0.15 [0.95–1.13]	T_20_ = −0.478	*d* = 0.166 (2%)
	♂	1.06 ± 0.05 [1.03–1.10]	*p* = 0.638	

Abbreviations: 1RM, one‐repetition maximum; BM, body mass; DPP_15s_, mean power during 15s double poling ergometer sprint; DPP_PEAK_, peak power during the double poling ergometry incremental test to exhaustion; FFM, fat‐free mass; *P*_max_, peak power during treadmill running; *v*La_max_, maximal lactate accumulation rate; VO_2max_, maximal oxygen uptake; VO_2peak_‐DP, peak oxygen uptake during double poling ergometry.

*p < 0.05.

ΔVO_2max_‐VO_2peak_, both absolute (male, 659 ± 374 [372–947] mL·min^−1^ vs. female, 472 ± 195 [354–590] mL·min^−1^, *p* = 0.139) and normalized to BM (male, 10.0 ± 5.0 [6.2–13.8] mL kg^−1^ min^−1^ vs. female, 8.9 ± 3.1 [7.0–10.8] mL kg^−1^ min^−1^, *p* = 0.532) or FFM (male, 11.5 ± 5.4 [7.3–15.7] mL·kg^−1^·min^−1^ vs. female, 10,6 ± 5.2 [7.4–13.7] mL·kg^−1^·min^−1^, *p* = 0.690), did not differ between male and female participants. When expressed relatively as the ratio of VO_2peak_ · VO_2max_^−1^, the differences did not differ between sexes (male, 0.85 ± 0.07 [0.79–0.90] vs. female, 0.84 ± 0.06 [0.81–0.88], *p* = 0.855).

The study found significant correlations between absolute VO_2max_ and absolute VO_2peak_‐DP (*r* = 0.93, *p* < 0.01), remaining robust after adjusting for sex (*r* = 0.87, *p* < 0.01). The correlations between these variables normalized to BM (*r* = 0.81, *p* < 0.01) and FFM (*r* = 0.91, *p* < 0.01) exhibited significant correlations, which persisted after sex correction (*r* = 0.70 and *r* = 0.90, both *p* < 0.01). FFM showed significant correlations with absolute VO_2peak_‐DP (rxy = 0.86; rxy‐z = 0.71, both *p* < 0.01), *v*Lamax (rxy = 0.53, *p* < 0.01; rxy‐z = 0.58, *p* < 0.01), and 1RM (rxy = 0.83–0.90; rxy‐z = 0.73–85, both *p* < 0.01). Tables 3 and 4 display the correlations between maximal upper‐body strength and endurance capacities and DP performance.

**TABLE 3:** Correlations (*r*‐values) between different physiological indices during maximal endurance tests, as well as upper‐body anaerobic capacity tests and performance (power output during double poling ergometry) in a group of male (*n* = 9) and female (*n* = 13) elite youth cross‐country skiing and biathlon athletes.

	VO_2max_	VO_2max_·BM^−1^	VO_2max_·FFM^−1^	VO_2peak_‐DP	VO_2peak_‐DP·BM^−1^	VO_2peak_‐DP·FFM^−1^	*v*La_max_
	(mL·min^−1^)	(mL·kg^−1^ min^−1^)	(mL·kg^−1^ min^−1^)	(mL·min^−1^)	(mL·kg^−1^ min^−1^)	(mL·kg^−1^ min^−1^)	(mmol·L^−1^ s^−1^)
DPP_PEAK_ (W)							
R_xy_	0.86** [0.74 to 0.98]	0.57** [0.27 to 0.87]	0.05 [−0.54 to 0.64]	0.87** [0.76 to 0.98]	0.44** [0.08 to 0.80]	0.04 [−0.41 to 0.49]	0.72** [0.50 to 0.94]
R_xy‐z_	0.76** [0.57 to 0.95]	0.24 [−0.18 to 0.66]	−0.21 [−0.77 to 0.36]	0.76** [0.57 to 0.95]	0.11 [−0.33 to 0.55]	−0.22 [0.78 to 0.34]	0.80** [0.64 to 0.96]
DPP_PEAK_·BM^−1^ (W·kg^−1^)							
R_xy_	0.53** [0.21 to 0.85]	0.55** [0.24 to 0.86]	−0.12 [−0.70 to 0.46]	0.65** [0.37 to 1]	0.61** [0.39 to 0.91]	−0.14 [−0.72 to 0.44]	0.50** [0.16 to 0.84]
R_xy‐z_	0.23 [−0.2 to 0.66]	0.26 [−0.16 to 0.68]	−0.40* [−0.90 to 0.10]	0.41* [0.04 to 0.78]	0.42* [0.05 to 0.79]	−0.42 [−0.91 to 0.07]	0.50** [0.16 to 0.84]
DPP_PEAK_·FFM^−1^ (W·kg^−1^)							
R_xy_	0.26 [−0.16 to 0.68]	0.15 [−0.43 to 0.73]	0.83** [0.65 to 1.00]	0.16 [−0.28 to 0.60]	−0.05 [−0.64 to 0.54]	0.83** [0.65 to 1.00]	0.18 [−0.75 to 0.39]
R_xy‐z_	0.22 [‐0.34 to 0.78]	0.06 [‐0.53 to 0.65]	0.84** [0.71 to 0.97]	0.08 [‐0.51 to 0.67]	−0.17 [‐0.74 to 0.40]	0.84** [0.71 to 0.97]	0.16 [‐0.28 to 0.60]
DPP_15s_ (W)							
R_xy_	0.80** [0.64 to 0.96]	0.38* [0.00 to 0.76]	0.20 [−0.23 to 0.63]	0.74** [0.54 to 0.94]	0.20 [−0.23 to 0.63]	−0.16 [−0.60 to 0.28]	0.81** [0.66 to 0.96]
R_xy‐z_	0.71** [0.49 to 0.93]	0.04 [−0.41 to 0.49]	0.04 [−0.41 to 0.49]	0.61** [0.33 to 0.89]	−0.16 [−0.60 to 0.28]	−0.00 [−0.59 to 0.59]	0.85** [0.73 to 0.97]
DPP_15s_·BM^−1^ (W·kg^−1^)							
R_xy_	0.59** [0.30 to 0.88]	0.37* [−0.02 to 0.76]	0.15 [−0.43 to 0.73]	0.58** [0.28 to 0.88]	0.28 [−0.13 to 0.69]	0.07 [−0.52 to 0.66]	0.74** [0.54 to 0.94]
R_xy‐z_	0.38* [0.00 to 0.76]	0.04 [−0.41 to 0.49]	−0.02 [−0.61 to 0.57]	0.35* [−0.04 to 0.74]	−0.03 [−0.48 to 0.42]	−0.11 [−0.69 to 0.47]	0.76** [0.57 to 0.95]
DPP_15s_·FFM^−1^ (W·kg^−1^)							
R_xy_	0.29 [−0.25 to 0.83]	0.24 [−0.18 to 0.66]	0.77** [0.53 to 1]	0.23 [−0.2 to 0.66]	0.09 [−0.50 to 0.68]	0.69** [0.38 to 1.00]	0.22 [−0.21 to 0.65]
R_xy‐z_	0.28 [−0.13 to 0.69]	0.20 [−0.23 to 0.63]	0.77** [0.53 to 1]	0.19 [−0.38 to 0.76]	0.02 [−0.57 to 0.61]	0.69** [0.38 to 1.00]	0.20 [−0.23 to 0.63]

*Note:* *R*‐values interpreted as: < 0.1, trivial (light red); > 0.1–0.3, small (yellow); > 0.3–0.5, moderate (green); > 0.5, large (dark green).

Abbreviations: BM, body mass; DPP_15s_, mean power during 15s double poling ergometer sprint; DPP_PEAK_, peak power during the double poling ergometry incremental test to exhaustion; FFM, fat‐free mass; R_xy_, correlation coefficients not corrected for sex; R_xy‐z_, correlation coefficients corrected for sex; *v*La_max_, maximal lactate accumulation rate; VO_2peak_‐DP, peak oxygen uptake during double poling ergometry.

*p < 0.05. **p < 0.01.

**TABLE 4:** Correlations (*r*‐values) between different physiological indices during maximal strength tests, as well fat‐free mass and performance (power output during double poling ergometry) in a group of male (*n* = 9) and female (*n* = 13) elite youth cross‐country skiing and biathlon athletes.

	FFM	1RM bench pull	1RM·BM^−1^ bench pull	1RM·FFM^−1^ bench pull	1RM bench press	1RM·BM^−1^ bench press	1RM·FFM^−1^ bench press
	(kg)	(kg)	(kg·kg^−1^)	(kg·kg^−1^)	(kg)	(kg·kg^−1^)	(kg·kg^−1^)
DPP_PEAK_ (W)							
R_xy_	0.86** [0.74 to 0.98]	0.84** [0.71 to 0.97]	0.66** [0.41 to 0.91]	0.35* [−0.04 to 0.74]	0.76** [0.57 to 0.95]	0.52** [0.19 to 0.85]	0.40* [0.02 to 0.78]
R_xy‐z_	0.75** [0.55 to 0.95]	0.74** [0.54 to 0.94]	0.44* [0.08 to 0.80]	0.29 [−0.25 to 0.83]	0.61** [0.33 to 0.89]	0.27 [−0.28 to 0.82]	0.44* [0.08 to 0.80]
DPP_PEAK_·BM^−1^ (W·kg^−1^)							
R_xy_	0.51** [0.18 to 0.84]	0.49* [0.15 to 0.83]	0.55** [0.24 to 0.86]	0.20 [−0.23 to 0.63]	0.43* [0.06 to 0.80]	0.41* [0.04 to to 0.78]	0.26 [−0.16 to 0.68]
R_xy‐z_	0.16 [−0.28 to 0.60]	0.22 [−0.21 to 0.65]	0.30 [−0.11 to 0.71]	0.11 [−0.33 to 0.55]	0.13 [−0.31 to 0.57]	0.12 [−0.32 to 0.56]	0.24 [−0.18 to 0.66]
DPP_PEAK_·FFM^−1^ (W·kg^−1^)							
R_xy_	0.24 [−0.18 to 0.66]	0.30 [−0.24 to 0.84]	0.25 [−0.30 to 0.80]	0.12 [−0.32 to 0.56]	0.23 [−0.2 to 0.66]	0.14 [−0.44 to 0.72]	0.26 [−0.16 to 0.68]
R_xy‐z_	0.19 [−0.38 to 0.76]	0.26 [−0.16 to 0.68]	0.20 [−0.23 to 0.63]	0.10 [−0.49 to 0.69]	0.17 [−0.40 to 0.74]	0.07 [−0.52 to 0.66]	0.25 [−0.30 to 0.80]
DPP_15s_ (W)							
R_xy_	0.84** [0.71 to 0.97]	0.88** [0.76 to 0.98]	0.65** [0.39 to 0.91]	0.49** [0.15 to 0.83]	0.82** [0.67 to 0.97]	0.58** [0.28 to 0.88]	0.51** [0.18 to 0.84]
R_xy‐z_	0.78** [0.60 to 0.96]	0.82** [0.67 to 0.97]	0.49** [0.15 to 0.83]	0.46* [−0.01 to 0.93]	0.74** [0.54 to 0.94]	0.40* [0.02 to 0.78]	0.53** [0.21 to 0.85]
DPP_15s_·BM^−1^ (W·kg^−1^)							
R_xy_	0.62** [0.34 to 0.90]	0.68** [0.44 to 0.92]	0.64** [0.37 to 0.91]	0.48** [0.13 to 0.83]	0.66** [0.41 to 0.91]	0.58** [0.28 to 0.88]	0.48** [0.13 to 0.83]
R_xy‐z_	0.42* [0.05 to 0.79]	0.54** [0.22 to 0.86]	0.48** [0.13 to 0.83]	0.45* [‐0.02 to 0.92]	0.52** [0.19 to 0.85]	0.41* [0.04 to 0.78]	0.50** [0.16 to 0.84]
DPP_15s_·FFM^−1^ (W·kg^−1^)							
R_xy_	0.22 [−0.21 to 0.65]	0.23 [−0.2 to 0.66]	0.15 [−0.43 to 0.73]	0.10 [−0.49 to 0.69]	0.19 [−0.38 to 0.76]	0.11 [−0.47 to 0.69]	0.15 [−0.43 to 0.73]
R_xy‐z_	0.17 [−0.40 to 0.74]	0.19 [−0.38 to 0.76]	0.09 [−0.50 to 0.68]	0.07 [−0.52 to 0.66]	0.14 [−0.44 to 0.72]	0.04 [−0.41 to 0.49]	0.14 [−0.44 to 0.72]

*Note:* *R*‐values interpreted as: < 0.1, trivial (light red); > 0.1–0.3, small (yellow); > 0.3–0.5, moderate (green); > 0.5, large (dark green).

Abbreviations: 1RM, one‐repetition maximum; BM, body mass; DPP_15s_, mean power during 15s double poling ergometer sprint; DPP_PEAK_, peak power during the double poling ergometry incremental test to exhaustion; FFM, fat‐free mass; R_xy_, correlation coefficients not corrected for sex; R_xy‐z_, correlation coefficients corrected for sex.

*p < 0.05. **p < 0.01.

## 4 Discussion

This is the first study to compare the physiological and performance characteristics of youth elite male and female cross‐country skiers and biathletes during DP ergometry. The findings demonstrated that when DP ergometry performances were normalized for FFM, the discrepancies between male and female athletes were largely eliminated. Moreover, the correlation coefficients between absolute and FFM normalized physiological indices and performance measures remained consistent even after correction for sex, suggesting that sex performance differences are primarily attributable to absolute differences in maximal aerobic power, maximal strength, and FFM.

The present study revealed significant sex differences in DP ergometer performances, with males exhibiting markedly higher absolute power production compared to females (*d* = 1.623–1.217, 31%–33%). These differences remained significant even after normalization to BM (*d* = 1.391–1.173, 16%–18%) but were eliminated through normalization to FFM (*d* = 0.263–0.298, 7%). Prior research has indicated that absolute upper‐body performance among adult male and female national‐level and elite cross‐country skiers exhibits even more pronounced sex differences, with the discrepancies ranging from 67% to 108% (Sandbakk, Ettema, and Holmberg 2014b; Hegge et al. 2015, 2016). However, the reported differences were reduced to values approaching those presented in the current study when DP power was normalized to BM (20%) or FFM (12%) (Sandbakk, Ettema, and Holmberg 2014b). In another study, the normalization of power output to lean upper‐body mass resulted in a reduction of the observed sex differences from 95% to 108% to only 37%–48% (Hegge et al. 2015). The discrepancy in power production between the sexes has been linked to the greater upper‐body muscle mass commonly observed in males by previous research (Sandbakk, Solli, and Holmberg 2018; Hegge et al. 2016; Janssen et al. 2000). It is noteworthy that the differences in BM and FFM between male and female participants in this study (*d* = , 1.036, 14% and *d* = 2.060, 29%, respectively) were less pronounced than those observed in previous research (*d* = 1.989–3.816, 20%–38% and *d* = 3.302–5.158, 34%–48%, respectively). This discrepancy is likely attributable to the younger age of the participants and the more mature physical development of the female youth athletes, compared to their male counterparts. During puberty, males and females undergo distinct physiological changes in which the increase in testosterone levels among males leads to higher hemoglobin concentrations, greater muscle mass, and lower‐body fat, whereas the same increase does not occur in females. These changes exaggerate sex differences in the delivery of aerobic and anaerobic energy and maximal strength, leading to an increased performance gap between sexes, even when adjusted for relative performance levels (Sandbakk, Solli, and Holmberg 2018; Joyner 2017). This physiological advantage is particularly pronounced in upper‐body‐intensive activities like DP. A study by Sollie and Losnegard (Sollie and Losnegard 2022), which was specifically designed to examine treadmill roller‐ski skating using the G2 sub‐technique, revealed that, as athletes progress through adolescence, sex differences in performance increase until they reach a plateau on levels that are comparable to those observed in elite adult athletes. However, as the present study did not collect data on maturity status, hormonal levels, or physiological maturity, any discussion of these influences must remain speculative. Further research is needed to accurately assess the impact of physiological maturity on performance.

The evident discrepancy in DP power between the sexes coincided with a considerable divergence in VO_2peak_‐DP (*d* = 1.985, 29%), which exhibited a notable reduction through normalization to FFM (*d* = 0.643, 12%). Similar observations have been reported in earlier studies among older and higher‐performing athletes, however, with pronounced disparities between sexes that remained significant even after normalization (Sandbakk, Ettema, and Holmberg 2014b; Sunde et al. 2019; Hegge et al. 2015, 2016). In contrast, no sex‐specific differences in the fractional utilization of VO_2max_ during DP were identified, which aligns with the findings of previous studies (Wagner et al. 2024; Hansen et al. 2021). These findings indicate that the upper body exhibits comparable oxidative capacity between sexes, with differences in VO_2peak_‐DP potentially attributable to variations in active muscle mass and/or peak oxygen transport capacity, in which the stroke volume is regarded as the primary determinant (Hegge et al. 2016; Joyner 2017). Research in older, higher‐performing athletes indicates that males exhibit smaller differences in peak oxygen uptake between upper‐ and lower‐body exercise modalities compared to females, supporting the notion of an increasing performance gap between sexes with age and performance level (Sandbakk, Ettema, and Holmberg 2014b; Sandbakk, Solli, and Holmberg 2018; Hegge et al. 2015). However, previous researched has demonstrated that both VO_2peak_‐DP and the fractional utilization of VO_2peak_‐DP can be enhanced through upper‐body‐specific training modalities, irrespective of sex (Wagner et al. 2024; Terzis, Stattin, and Holmberg 2006). Consequently, while the endurance performance gap between sexes is primarily associated with significant differences in VO_2max_, these findings prompt consideration as to whether certain previously observed sex differences, such as fractional utilization of VO_2max_ during DP, are solely innate or partially influenced by disparities in training regimes.

Correlations of absolute VO_2max_ and VO_2peak_‐DP with power output during DP ergometry were significant (*r*_xy_ = 0.74–0.87) and remained consistent even after correction for sex (*r*_xy‐z_ = 0.61–0.76). These findings reinforce the critical role of absolute aerobic power in determining DP performance outcomes, regardless of sex. However, the correlation between BM‐normalized oxygen uptake measures and power metrics was found to be relatively low, particularly when adjusted for sex (see Table 3). Similar correlations were identified between VO_2peak_‐DP and 800‐m and 100‐m performances using a DP ergometer in a previous study (*r* = −0.61 to −0.86) (Støren et al. 2023), which aligns with other research linking peak aerobic power and ski racing performance more generally (*r* = |0.41| to |0.90|) (Alsobrook et al. 2009; Carlsson et al. 2016). It is noteworthy that FFM‐normalized measures of oxygen uptake and DP performance exhibited similar correlations as absolute measures, which remained unaltered by the correction for sex. This provides further evidence that active muscle mass plays a significant role in determining performance, as supported by the significant correlations between FFM and DP performances observed in this and previous studies (Carlsson et al., 2014). Although body weight‐normalized values are commonly used to facilitate comparisons between individuals of varying body sizes, this relative measure may not fully account for the factors influencing power output during DP ergometry, such as absolute (aerobic) power and active muscle mass. This may be particularly relevant in the context of upper‐body dominant techniques or in short‐distance events, where absolute power is of paramount importance (Sandbakk, Ettema, and Holmberg 2014b; Sandbakk, Solli, and Holmberg 2018). The data from this study indicate that enhancing absolute VO_2max_ and VO_2peak_‐DP, as well as increasing upper‐body muscle mass, may prove to be a more beneficial strategy for enhancing power output than focusing solely on weight‐normalized VO_2max_ and VO_2peak_‐DP through the reduction of BM.

In the case of *v*Lamax, a proposed estimation of the maximum rate of glycolysis (Heck, Schulz, and Bartmus 2003), no significant difference between sexes was identified, despite the presence of variations in BM and FFM. This finding is consistent with prior literature indicating no sex disparity across different age groups for markers of anaerobic capacity (Sollie and Losnegard 2022). These findings are inconsistent with the proposed association between elevated lactate levels (i.e., anaerobic capacity) and a greater proportion of active muscle mass and fast glycolytic muscle fibers (Perez‐Gomez et al. 2008; Ivy et al. 1980). This discrepancy may be attributed to the complex nature of blood lactate concentration, which is influenced by various factors related to lactate production, distribution, and clearance (Brooks 2018). These complexities present significant methodological challenges in testing anaerobic capacity, as the testing requires exercise efforts that maximize glycolysis while minimizing the activation of oxidative metabolism to reduce intramuscular oxidation. Nevertheless, the robust correlations between *v*Lamax and DPP15s, both in absolute and BM‐normalized terms, support previous findings that anaerobic metabolism contributes substantially to power production (up to 20%–30%), particularly during high‐intensity efforts and short‐duration events (Haugen et al. 2021).

The present study identified significant differences in 1RM between the sexes (*d* = 1.395–1.443, 31%–36%), which diminished through normalization for FFM (*d* = 0.166–0.342, 2%–6%), a finding that is consistent with previous research (Sollie and Losnegard 2022; Perez‐Gomez et al. 2008). However, previous literature has indicated that sex differences in maximal strength among national‐ and international‐level cross‐country skiers are markedly greater (*d* = 3.873, 58%) and are associated with more pronounced sex differences in DP performance. These findings highlight the critical role of a high maximal strength level in achieving superior cross‐country skiing performance. In this sample, significant correlations were identified between the 1RM and both aerobic (DPP_PEAK_, *r* = 0.41–0.84) and anaerobic (DPP_15s_, *r* = 0.51–0.87) performance, with stronger correlations observed for absolute values. It is noteworthy that, upon correction for sex, most correlations remained significant but exhibited a slight reduction in correlative strength. Furthermore, normalizing to FFM eliminated the differences in 1RM and DP performances to the extent that no significant correlations could be found. The same phenomenon also prevented significant correlations between *v*Lamax and FFM normalized DP performances. Given that strength and power are associated with superior performance in cross‐country skiing in general (Stoggl et al. 2011; Sunde et al. 2019; Alsobrook et al. 2009), these results may be particularly relevant for female athletes, as muscle mass and strength are trainable factors in both sexes, and females have previously been reported to have strength deficits compared to their male counterparts (Stoggl et al. 2011). However, cross‐country skiing performance is not solely dependent on maximum strength but also on the precise timing and coordination of force application. There is a potential maximal strength threshold beyond which further gains do not enhance performance. This underscores the importance of strength as a foundation for proper skiing technique, which in turn may contribute to enhanced skiing performance (Stoggl et al. 2011).

The findings of this study provide valuable insights, although it is essential to acknowledge the limitations of the research design. The use of a convenience sample introduces the potential for bias, including sampling bias, self‐selection bias, and observer bias, which may result in a nonstatistically balanced selection. As the sample was not randomly selected, it is unlikely to be fully representative of the population, which limits the generalizability of the results. Furthermore, the limited sample size, a common issue in elite sports research, resulted in a smaller number of participants per sex, thereby limiting the statistical power of the study. A small sample size increases the probability that a single outlier could exert a significant influence on the data, thereby introducing bias and further compromising the results. Accordingly, the findings of this study should be interpreted with a certain degree of caution. A comprehensive classification in existing literature is essential for accurate conclusions to be drawn. To quantify FFM, bioimpedance analysis was utilized. Although this method is not considered the gold standard, previous literature has demonstrated its reliability, despite a systematic measurement error that leads to a general overestimation of FFM compared to dual x‐ray absorptiometry, which is considered the current gold standard (McLester et al. 2020). Nevertheless, it allows for valid group comparisons. Notwithstanding these limitations, the data collected remains valuable, offering unique insights into a seldom‐studied athletic group, thus contributing to the advancement of knowledge in the field of high‐performance sports.

## 5 Conclusions

This study on youth elite cross‐country skiers and biathletes found sex disparities in the DP performance of 33%, 17%–18%, and 3%–7% in absolute, body‐mass, and FFM normalized power values, respectively, which are lower than previously found for adult skiers in absolute power but similar when normalized for BM. Given that these differences seem particularly driven by the combination of maximal aerobic power muscle mass and strength characteristics, upper‐body‐specific strength and power training should be prioritized in athletes, particularly female athletes, who have less upper‐body muscle mass. Recent research showed that high‐volume resistance training could induce enlargement in upper‐body musculature that subsequently increases VO_2peak_ and power output during DP (Wagner et al. 2024). Consequently, recognizing that male and female athletes experience different physiological developments, coaches need to consider adapting training programs accordingly to support physiological changes and minimize sex differences. As differences in upper‐body strength, power, and aerobic power may also result from varying emphases on upper‐body strength and endurance training, future research should investigate the extent to which systematic upper‐body training can mitigate these disparities. This would provide a clearer understanding of how to optimize training protocols for both male and female athletes.

## Ethics Statement

This study was performed in accordance with the Declaration of Helsinki and was approved by the Universities Ethics Committee (DHGS‐EK‐2023‐004).

## Consent

Each subject and their parents (if the subjects were younger than 18 years) were informed about the aims of the study and the experimental risks involved with the research before providing written informed consent.

## Conflicts of Interest

The authors declare no conflicts of interest.

## Data Availability

The data that support the findings of this study are available from the corresponding author upon reasonable request.
